# Blind Estimation of the PN Sequence of A DSSS Signal Using A Modified Online Unsupervised Learning Machine

**DOI:** 10.3390/s19020354

**Published:** 2019-01-16

**Authors:** Yangjie Wei, Shiliang Fang, Xiaoyan Wang, Shuxia Huang

**Affiliations:** Key Laboratory of Underwater Acoustic Signal Processing of Ministry of Education, Southeast University, Nanjing 210096, China; xyan@seu.edu.cn (X.W.); 13404133160@163.com (S.H.)

**Keywords:** PN sequence estimation, DSSS signals, PCA, modified LEAP

## Abstract

Direct sequence spread spectrum (DSSS) signals are now widely used in air and underwater acoustic communications. A receiver which does not know the pseudo-random (PN) sequence cannot demodulate the DSSS signal. In this paper, firstly, the principle of principal component analysis (PCA) for PN sequence estimation of the DSSS signal is analyzed, then a modified online unsupervised learning machine (LEAP) is introduced for PCA. Compared with the original LEAP, the modified LEAP has the following improvements: (1) By normalizing the system state transition matrices, the modified LEAP can obtain better robustness when the training errors occur; (2) with using variable learning steps instead of a fixed one, the modified LEAP not only converges faster but also has excellent estimation performance. When the modified LEAP is converging, we can utilize the network connection weights which are the eigenvectors of the autocorrelation matrix of the DSSS signal to estimate the PN sequence. Due to the phase ambiguity of the eigenvectors, a novel approach which is based on the properties of the PN sequence is proposed here to exclude the wrong estimated PN sequences. Simulation results showed that the methods mentioned above can estimate the PN sequence rapidly and robustly, even when the DSSS signal is far below the noise level.

## 1. Introduction

Direct sequence spread spectrum (DSSS) signals have been used widely in military as well as civil communication systems for several decades because of their strong anti-interference ability and low probability of interception property [1,2,3,4,5,6,7]. In a non-cooperative DSSS communication system, the pseudo-random (PN) sequence of the DSSS signal is a crucial parameter for blind dispreading. Consequently, it is of great significance to estimate it. Many methods for PN sequence estimation of DSSS signals have been published in recent years [8,9,10,11,12,13,14,15,16,17,18,19,20].

In Reference [8], Warner et al. firstly used triple correlation function (TCF) to estimate the spreading code; since then, many algorithms based on it have been proposed for PN sequence estimation [9,10,11,12,13]. These TCF-based methods can be used not only for PN sequence estimation, but also for detecting DSSS signals. They can work well in good conditions, but as the environment gets worse, the performance of these algorithms deteriorates sharply. 

In Reference [14], Burel et al. introduced an algorithm to PN sequence estimation of the DSSS signal based on eigenvalue decomposition (EVD). In this method, the received DSSS signal is sampled and divided into temporal windows, the size of which is the PN sequence period. Each window provides a vector for the eigenanalysis, then the PN sequence can be estimated by the eigenvectors. In Reference [15], the sampled DSSS signal was divided into continuous non-overlapping temporal vectors with a width of two periods of PN sequence; then, the PN sequence could be estimated by employing the EVD method after the average correlation matrix was calculated. In Reference [16], Qui et al. proposed a PN sequence estimation method. In this method, the signal was divided into a series of overlapping windows with the width much shorter than the information symbol width, and then the segments of the spreading code were estimated using the EVD method and a complete spreading code was obtained from the estimated segments. Although these EVD based methods [14,15,16,17,18] show excellent performance in low signal to noise ratio (SNR) conditions, they become expensive in terms of computing when the length of the PN sequence is long. In order to solve this problem, many PN sequence estimation methods based on neural network have been proposed. 

For example, in Reference [19], Dominique et al. introduced a subspace-based PN sequence estimation algorithm for DSSS signals using a simplified Hebb rule, which can reduce the number of computations required compared to the existing Hebb-based sequence estimator. The main advantage of these Hebb estimators is that the estimator architecture is very simple and can be implemented easily, but the constant small learning steps severely limit their performance.

In Reference [20], Chen et al. proposed a modified online supervised learning machine (LEAP) to extract multiple principal components. In other words, the LEAP can be used for principal component analysis (PCA). This algorithm is adaptive to nonstationary input and requires no knowledge of, or when, the input changes statistically. Since it requires little memory or data storage, the LEAP is very suitable for use in engineering. However, the constant small learning step also severely limits its performance. Although the convergence speed of the LEAP can be accelerated when using a large learning step, the LEAP often fails to converge to the global optimum point and the large learning step may damage the stability of the system. Based on these mentioned above, in this paper, we propose a modified LEAP algorithm and apply it into the PN sequence estimation of the DSSS signal. Compared to the original LEAP, the modified LEAP uses variable learning steps instead of a fixed one, which can greatly improve the convergence performance of the network. Namely, the modified LEAP first makes the network close to the optimum convergence point with large learning steps when the network starts training, then when the network approaches the optimum convergence point, small learning steps are used, thus ensuring the network converges to the best point. Meanwhile, in order to maintain the stability of the network when using variable learning steps, the state transition matrices of the system are normalized. In summary, our main contributions lie in the following folds:

(a) The LEAP algorithm is applied into the field of the PN sequence estimation of DSSS signals and a modified LEAP algorithm is proposed. Compared to the original LEAP algorithm, the modified LEAP algorithm has a better convergence performance due to its use of variable learning steps rather than a fixed one;
(b) Since the phase of the eigenvector can be inverted, the incorrect estimation of the PN sequence of the DSSS signal may be obtained. Based on this, a novel approach which makes full use of the correlation characteristics of the PN sequence is proposed here to solve this problem.

This paper is organized as follows. In Section 2, firstly, the mathematical model of DSSS signals and the principle of PCA for PN sequence estimation are given, then a modified LEAP is introduced. The method for PN sequence estimation and the elimination of phase ambiguity are described in Section 3. The main steps for PN sequence estimation of DSSS signals are described in Section 4. Simulation results are presented in Section 5. Finally, a conclusion is drawn in Section 6.

## 2. Basic Theories

### 2.1. DSSS Signal Model

In a DSSS transmission, the symbols are multiplied by a PN sequence, which spreads the bandwidth. In this paper, we use the notations below [14]:

p(t): The convolution of the transmission filter, the channel filter (which represents the channel echoes) and the receiver filter. 

$\left\{c_{m},m=0,1,2,\cdots ,N-1\right\}$: The PN sequence.

*N*: The length of the PN sequence.

$T_{p}$: The symbol period.

$T_{c}$: The chip period ($T_{c}=T_{p}/N$).

h(t): The convolution of the PN sequence with all the filters of the transmission chain (transmitter filter, channel echoes, and receiver filter):

(1) $$h\left(t\right)=\sum_{m=0}^{N-1}c_{m}p\left(t-mT_{c}\right).$$

*h*: The vector containing the samples of h(t).

s(t): The DSSS baseband signal at the output of the receiver filter:

(2) $$s\left(t\right)=\sum_{l=-\infty }^{+\infty }a_{l}h\left(t-lT_{p}\right).$$

$a_{l}$: The message symbols.

v(t): The noise at the output of the receiver filter, which is uncorrelated with the signal.

Then, the baseband signal at the output of the receiver filter can be written as:

(3) x(t)=s(t)+v(t).

### 2.2. The Principle of PCA for PN Sequence Estimation

Since many algorithms can estimate $T_{p}$ and $T_{c}$, they are assumed to be obtained in advance in this paper [15]. After being sampled (the sampling rate is $1/T_{c}$ ) and passed through an observation window with duration $T_{p}$, the received DSSS signal x(t) can produce a series of observed sample vectors after each interval $T_{p}$. Let us note x(k) the content of a window, then the x(k) can be modeled as:

(4) x(k)=s(k)+v(k),k=1,2,3,⋯,

where the dimension of x(k) is $N=T_{p}/T_{c}$, k represents the discrete time. Usually, the observation widow has a random time delay $T_{x}$, which is the desynchronization between windows and symbols $\left(0\leq T_{x}<T_{p}\right)$. Therefore, s(k) generally contains two consecutive message symbol bits, and s(k) can be written as:

(5) $$s\left(k\right)=a_{k}h_{1}+a_{k+1}h_{2},$$

where $a_{k}$, $a_{k+1}$ are the two consecutive message symbols. 

$h_{1}$ is a vector containing the end (duration $T_{p}-T_{c}$) of the spreading waveform h(t), followed by zeros (duration $T_{x}$).

$h_{2}$ is a vector containing the zeros (duration $T_{p}-T_{x}$) followed by the beginning (duration $T_{x}$) of the spreading waveform h(t).

Let $e_{i}=h_{i}/\|h_{i}\|,i=1,2$, we can obtain:

(6) $$e_{i}^{T}e_{j}=\delta \left(i-j\right),\left(i,j=1,2\right),$$

where $e_{i},i=1,2$ are orthonormalized vectors and δ(·) is the Dirac function. Then, the x(k) can be expressed as follows:

(7) $$x\left(k\right)=a_{k}\left\|h_{1}\right\|e_{1}+a_{k+1}\left\|h_{2}\right\|e_{2}+v\left(k\right).$$

Using the equations above, the autocorrelation matrix $R_{x}$ of the DSSS signal can be obtained by:

(8) $$R_{x}=E\left[xx^{T}\right]=\left[\sigma_{n}^{2}\eta \frac{T_{p}-T_{x}}{T_{c}}\right]e_{1}e_{1}^{T}+\left(\sigma_{n}^{2}\eta \frac{T_{x}}{T_{c}}\right)e_{2}e_{2}^{T}+\sigma_{n}^{2}I,$$

where E{·} denotes expectation, $\sigma_{n\;}^{2}$ is the variance of the noise, $\eta =\sigma_{s\;}^{2}/\sigma_{n\;}^{2}$, $\sigma_{s\;}^{2}$ is the variance of s(k), and I is an identity matrix of dimension N×N. From Equation (8), it is clear that two eigenvalues will be larger than the others when $T_{x}>0$, and according to their corresponding eigenvectors, which can be obtained by PCA, the PN sequence can be estimated.

### 2.3. Mathematical Model of The Modified LEAP

**Figure 1:**
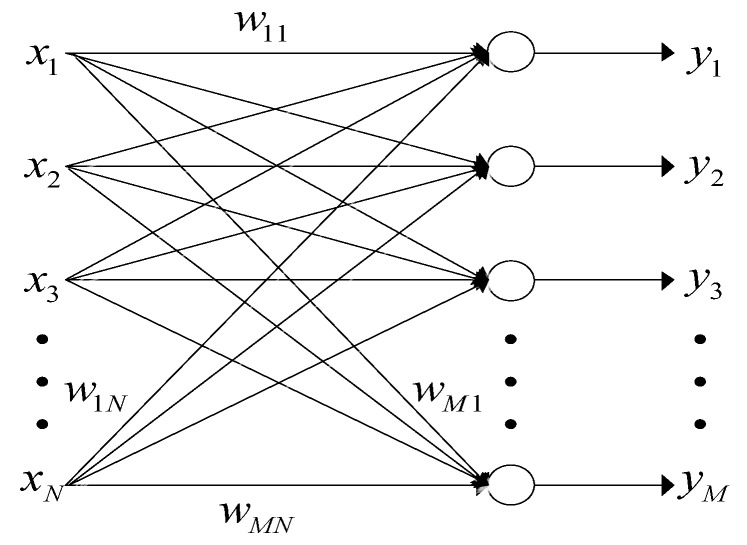
A neural network model of LEAP, an online unsupervised learning machine.

The LEAP is implemented on a neural network with linear units shown in Figure 1. Specifically, in many practical applications, M≪N. Let x(k) denote the input vector process, then the network’s input–output relation can be written as [20]:

(9) $$y_{i}\left(k\right)=w_{i}^{T}\left(k\right)x\left(k\right),i=1,2,\cdots ,M,$$

where $w_{i}\left(k\right)={\left[w_{i1}\left(k\right),w_{i2}\left(k\right),\cdots ,w_{iN}\left(k\right)\right]}^{T}$, for i=1,2,⋯,M. $x\left(k\right)={\left[x_{1}\left(k\right),x_{2}\left(k\right),\cdots ,x_{N}\left(k\right)\right]}^{T}$, $y\left(k\right)={\left[y_{1}\left(k\right),y_{2}\left(k\right),\cdots ,y_{M}\left(k\right)\right]}^{T}$. Here, $x_{i}\left(k\right)$ is the *i*th input of the network, $y_{j}\left(k\right)$ is the *j*th output, $w_{ij}\left(k\right)$ is the connection weight from the *j*th input to the *i*th output neuron, all at discrete time k. Supposing that $R_{x}$ has eigenvalues $\lambda_{1}>\lambda_{2}>\cdots >\lambda_{N}>0$ with corresponding normalized eigenvectors $e_{i,}i=1,2,\cdots ,N$, then $w_{i}\left(k\right)\to e_{i}$, $E\left\{y_{i}^{2}\right\}\to \lambda_{i}$, as k→∞, for i=1,2,⋯,M. 

The LEAP for connection weight updating is the following nonlinear non-autonomous dynamical vector difference equations:

(10) $$w_{i}\left(k+1\right)=w_{i}\left(k\right)+\beta \left\{B_{i}\left(k\right)\left[y_{i}\left(k\right)x\left(k\right)-y_{i}^{2}\left(k\right)w_{i}\left(k\right)\right]-A_{i}\left(k\right)w_{i}\left(k\right)\right\},$$

for i=1,2,⋯,M, and:

(11) $$A_{i}\left(k\right)=\{\begin{array}{ll} 0, & i=1\\ \sum_{j=1}^{i-1}w_{j}\left(k\right)w_{j}^{T}\left(k\right), & i=2,3,\cdots M \end{array}$$

(12) $$B_{i}\left(k\right)=I-A_{i}\left(k\right),i=1,2,\cdots ,M,$$

β is the constant learning step. 

In Equation (10), the $A_{i}$ and $B_{i}$ can be seen as the state transfer matrices of the system, which are important “de-correlation” terms for performing Gram–Schmidt orthogonalization among all connection weights at each iteration. One could think of the term $y_{i}x$ as the so-called Hebbian learning, for which the strengthening of the connection weights is proportional to the input–output correlation.

According to the theory in Reference [20], in the original LEAP, it is known that the learning step β should be small enough, otherwise the system performance can be severely degraded and training errors may occur, thus making the system unstable. Namely, there is an equilibrium point: If the learning step β exceeds this point, the system will be unusable. Therefore, in practice, the learning step is set as small as possible in the original LEAP, but it greatly increases the time required for network convergence. On the basis of the reasons above, a modified LEAP is proposed. First, the learning step β is modified to:

(13) $$\beta_{i}\left(k+1\right)=\alpha \beta_{i}\left(k\right)+\gamma \left(\left|\lambda_{i}\left(k\right)-\lambda_{i}\left(k-1\right)\right|/\max\left\{\lambda_{i}\left(k\right),\lambda_{i}\left(k-1\right)\right\}\right),$$

where:

(14) $$\lambda_{i}\left(k\right)=E\left\{y_{i}^{2}\right\},\;$$

for i=1,2,⋯,M, 0<α<1, γ>0, |·| denotes taking the absolute value, max{·} denotes taking the maximum value. Where α and γ are the weight coefficients in the variable learning steps and they are similar to the weight coefficients in the variable step size least mean square (LMS) algorithm. Compared to those of the original LEAP algorithm, the learning steps of the modified LEAP algorithm can be adaptively changed according to the output of the network. Namely, when the network starts training, the difference between $\lambda_{i}\left(k\right)$ and $\lambda_{i}\left(k-1\right)$ is large; the network uses large learning steps at this time, and when the network is about to converge, $\lambda_{i}\left(k\right)$ and $\lambda_{i}\left(k-1\right)$ are approximately equal, and the network uses small learning steps in this case. In this way, the modified LEAP can not only have fast convergence speed, but also get good steady-state performance. In Equation (13), the $\max\left\{\lambda_{i}\left(k\right),\lambda_{i}\left(k-1\right)\right\}$ is divided in the formula to prevent $\left|\lambda_{i}\left(k\right)-\lambda_{i}\left(k-1\right)\right|$ from becoming too large, which may be caused by the training errors etc., thus making the learning steps become too large and seriously affecting the convergence speed of the network. 

However, when the variable learning steps are used, since the learning step size of the network of the modified LEAP is slightly large at the beginning of the training, the step size may still exceed the equilibrium point mentioned in Reference [20]. At this time, the uncorrelation between the connection weights of the network may be destroyed, which results in the instability of the system. Therefore, the state transition matrix of the system is normalized here, that is:

(15) $$A_{i}\left(k\right)={A_{i}\left(k\right)/\left\|A_{i}\left(k\right)\right\|}_{F},i=2,3,\cdots ,M,$$

where ${\left\|\cdot \right\|}_{F}$ denotes the Frobenius norm. It is obvious that ${\left\|Ai(k)\right\|}_{F}$ is a compatible matrix norm, then $0<\lambda \left(A_{i}\left(k\right)\right)\leq 1$, $0<\lambda \left(B_{i}\left(k\right)\right)\leq 1$ and the normalization does not affect the decorrelation function of the matrices $A_{i}$, $B_{i}$. Therefore, according to the stability criterion of Liapunov [21,22], we can maintain the system stability, when the training errors damage the uncorrelation between the connection weights $w_{i}$.

### 2.4. Asymptotic Stability Analysis of The Modified LEAP

Since $\beta_{i}\left(k\right)$ is small when k→∞, and $A_{i}{\left(k\right)}_{F}\geq 1$, we can obtain this approximation:

(16) $$\frac{\beta_{i}\left(k\right)}{{\left\|A_{i}\left(k\right)\right\|}_{F}}\approx \beta_{i}\left(k\right),$$

for i=2,3,⋯,M. It can be easily shown that [20]:

(17) $$w_{i}\left(k\right)=e_{i},i=1,2,\cdots ,M$$

is an equilibrium point of Equation (10). Let $g_{i}\left(k\right)=w_{i}\left(k\right)-e_{i}$, for i=1,2,⋯,M, we can get the following approximations:

(18) $$g_{1}\left(k+1\right)-g_{1}\left(k\right)=\varsigma_{1}\left[R_{x}-\lambda_{1}\left(I+2e_{1}e_{1}^{T}\right)\right]g_{1}\left(k\right),\;$$

for i=1.

(19) $$\begin{array}{l} g_{i}\left(k+1\right)-g_{i}\left(k\right)\\ =\varsigma_{i}\{\left[R_{x}-\lambda_{i}\left(I+2e_{i}e_{i}^{T}\right)+\sum_{j=1}^{i-1}\left(\lambda_{i}-\lambda_{j}-1\right)e_{j}e_{j}^{T}\right]g_{i}(k),\\ -\sum_{j=1}^{i-1}e_{j}e_{i}^{T}g_{j}\left(k\right)\} \end{array}$$

for i=2,3,⋯,M. Here, $\varsigma_{i}=\mu \beta_{i}\left(k\right)$, μ is a positive integer. Then, Equations (18) and (19) can also be written as:

(20) $$\left[\begin{array}{c} g_{1}\left(k+1\right)\\ \begin{array}{l} g_{2}\left(k+1\right)\\ g_{3}\left(k+1\right) \end{array}\\ \vdots \\ g_{M}\left(k+1\right) \end{array}\right]=\left[\begin{array}{ccccc} I+\varsigma_{1}D_{1} & 0 & 0 & 0 & 0\\ -\varsigma_{2}e_{1}e_{2}^{T} & I+\varsigma_{2}D_{2} & 0 & 0 & 0\\ -\varsigma_{3}e_{1}e_{3}^{T} & -\varsigma_{3}e_{2}e_{3}^{T} & I+\varsigma_{3}D_{3} & 0 & 0\\ \vdots & \vdots & \vdots & \ddots & \vdots \\ -\varsigma_{M}e_{1}e_{M}^{T} & -\varsigma_{M}e_{2}e_{M}^{T} & -\varsigma_{M}e_{3}e_{M}^{T} & \cdots & I+\varsigma_{M}D_{M} \end{array}\right]\left[\begin{array}{c} g_{1}\left(k\right)\\ \begin{array}{l} g_{2}\left(k\right)\\ g_{3}\left(k\right) \end{array}\\ \vdots \\ g_{M}\left(k\right) \end{array}\right],$$

where:

(21) $$D_{i}=\{\begin{array}{l} R_{x}-\lambda_{1}\left(I+2e_{1}e_{1}^{T}\right),i=1\\ R_{x}-\lambda_{i}\left(I+2e_{i}e_{i}^{T}\right)+\sum_{j=1}^{i-1}\left(\lambda_{i}-\lambda_{j}-1\right)e_{j}e_{j}^{T},i=2,3,\cdots ,M \end{array}$$

and because:

(22) $$I=\sum_{i=1}^{N}e_{i}e_{i}^{T},$$

$D_{1}$ can be written as:

(23) $$D_{1}=\sum_{i=1}^{N}\lambda_{i}e_{i}e_{i}^{T}-\lambda_{1}\sum_{i=1}^{N}e_{i}e_{i}^{T}-2\lambda_{1}e_{1}e_{1}^{T}$$

According to Equation (23), it is obvious that $D_{1}$’s eigenvectors are $\left\{e_{1},e_{2},\cdots e_{N}\right\}$ with corresponding eigenvalues:

(24) $$\left\{-2\lambda_{1},\left(\lambda_{2}-\lambda_{1}\right),\cdots ,\left(\lambda_{N}-\lambda_{1}\right)\right\}.$$

Similarly, we can know that all $D_{i},i=2,3,\cdots ,M$ have the same eigenvectors $\left\{e_{1},e_{2},\cdots e_{N}\right\}$, and their corresponding eigenvalues are:

(25) $$\{\overset{i-1}{\overbrace{-1,\cdots ,-1,}}-2\lambda_{i},\overset{N-i}{\overbrace{\left(\lambda_{i+1}-\lambda_{i}),\cdots ,(\lambda_{N}-\lambda_{i}\right)}}\},$$

On the basis of Equations (24) and (25), it is clear that all $D_{i}$’s eigenvalues are negative. The magnitudes of all eigenvalues of $I+\varsigma_{i}\;D_{i}$ will be less than 1, if $\varsigma_{i}$ is small enough, for i=1,2,⋯,M. Therefore, the equilibrium point given by Equation (17) is asymptotically stable in the sense of Liapunov [21,22], i.e., there exists a neighborhood of the point that any solution initially in this neighborhood will converge to the equilibrium point as k→∞.

## 3. PN Sequence Estimation and The Elimination of Phase Ambiguity

When the modified LEAP is converging, which means:

(26) $$\left|\lambda_{i}\left(k\right)-\lambda_{i}\left(k-1\right)\right|<\varepsilon ,$$

where ε represents a threshold for judging whether the network is converging, 0<ε≪1, i=1,2,⋯M, then concatenating $e_{1}\left(k\right)$ and $e_{2}\left(k\right)$. Because the phase of eigenvectors can be inverted, four estimated sequences $b_{i},i=1,2,\cdots 4$ can be obtained. Then, the estimated PN sequence of the DSSS signal can be calculated by:

(27) $${PN}_{i}=\mathrm{sgn}\left(b_{i}\right),i=1,2,\cdots ,4,$$

where:

(28) $$\mathrm{sgn}\left(x\right)=\{\begin{array}{ll} 1, & x>0\\ 0, & x=0\\ -1, & x<0 \end{array}.$$

${\hat{PN}}_{i}$ is a vector of length N. In order to select the true estimated PN sequence from ${\hat{PN}}_{i},i=1,2,\cdots 4$, the correlation characteristics of the PN sequence of the DSSS signal can be used. Namely, the PN sequence has good autocorrelation and bad cross-correlation. Hence, let us define a correlation factor ψ:

(29) $$\psi_{i}=\sum_{\tau =0}^{N-1}\left|{\overset{⌢}{PN}}_{i}\left(n\right){\overset{⌢}{PN}}_{i}^{*}\left(n-\tau \right)\right|,$$

where τ=0,1,2,⋯N−1 denotes the discrete time delay and i=1,2,⋯4, * denotes the conjugate operation. Obviously, the smaller the ψ is, the worse the cross-correlation of the PN sequence is. Then, the correct index of the true estimated PN sequence among ${\hat{PN}}_{i},i=1,2,\cdots 4$ can be calculated by:

(30) $$id=\arg\underset{i=1,2,\cdots 4}{\min}\left[\psi_{i}\right],$$

where min{·} denotes taking the minimum value. Then, according to Equation (30), the true estimated PN sequence is ${\hat{PN}}_{id}$. In addition, in the absence of other prior information, the overall phase ambiguity of the ${\hat{PN}}_{id}$ cannot be eliminated, which means the true PN sequence could be either ${\hat{PN}}_{id}$ or $-{\hat{PN}}_{id}$. For example, if we know that this PN sequence is the m-sequences, then we can eliminate the overall phase ambiguity of the estimated PN sequence according to the equalization characteristics of the m-sequences; namely, in the m-sequences, the number of 1 is one more than the number of −1.

## 4. The Main Steps for PN Sequence Estimation

To be more specific, the main steps involved in this paper for PN sequence estimation of the DSSS signal are summarized as follows:

Step 1. Sample the received DSSS signal, then obtain x(k),k=1,2,3⋯. Meanwhile, in order to improve the system robustness, the neural network input x(k) should be normalized as follows:

(31) x(k)=x(k)/‖x(k)‖, 

where:

(32) $$\left\|x\left(k\right)\right\|=\sqrt{x^{T}\left(k\right)x\left(k\right)}.$$

Step 2. Setting the initial value of $\;w_{i},i=1,2,\cdots M$, which are often random numbers between −1 and 1, then normalizing:

(33) $$w_{i}=w_{i}/\left\|w_{i}\right\|,i=1,2,\cdots M.$$

Step 3. According to Equations (10)–(15), updating the weight vectors $\;w_{i},i=1,2,\cdots M$.

Step 4. Extracting the eigenvectors corresponding to the largest and second largest eigenvalues, when the neural network is converging. Subsequently, concatenating the two eigenvectors, then according to Equations (26)–(30), the PN sequence of the DSSS signal can be estimated. Then, the blind PN sequence estimation method of the DSSS signal proposed in this paper is derived.

## 5. Simulations and Analysis

**Figure 2:**
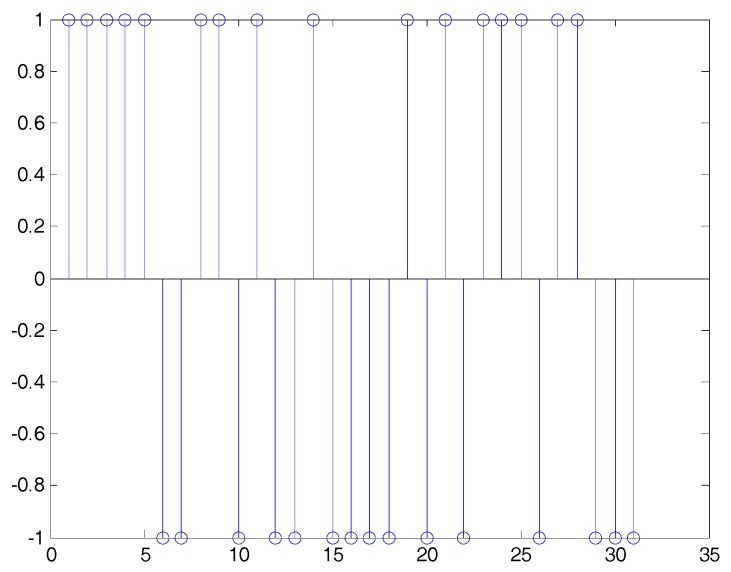
The true pseudo-random (PN) sequence of the direct sequence spread spectrum (DSSS) signal.

To verify the capability of the proposed method, simulation results are presented in this section. Here, the DSSS signal is generated using a random sequence of length 31 (it is one of the m-sequences); then, for completeness, we shall set N=M=31. The symbols belong to a BPSK constellation (binary phase shift keying). The noise is additive white Gaussian noise, which is uncorrelated with the DSSS signal. $T_{x}/T_{c}=10$. Figure 2 shows the true PN sequence of the DSSS signal. 

**Figure 3:**
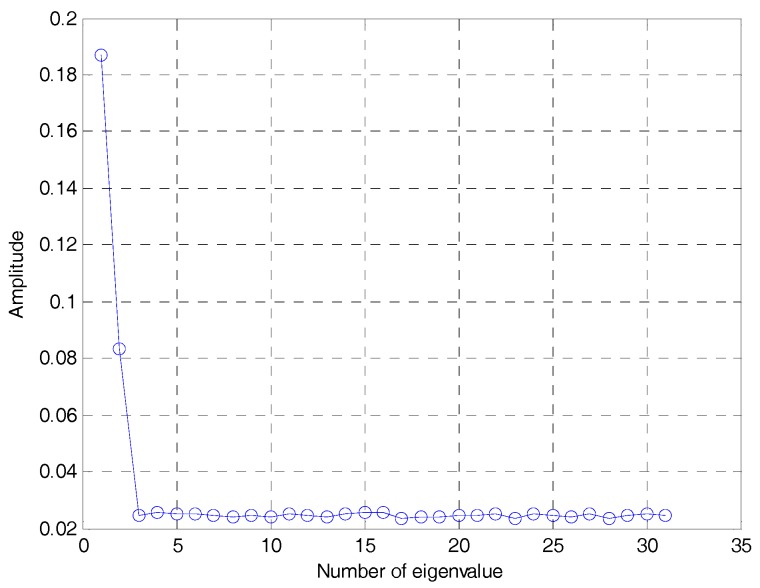
The estimated eigenvalues of the autocorrelation matrix of the DSSS signal.

**Figure 4:**
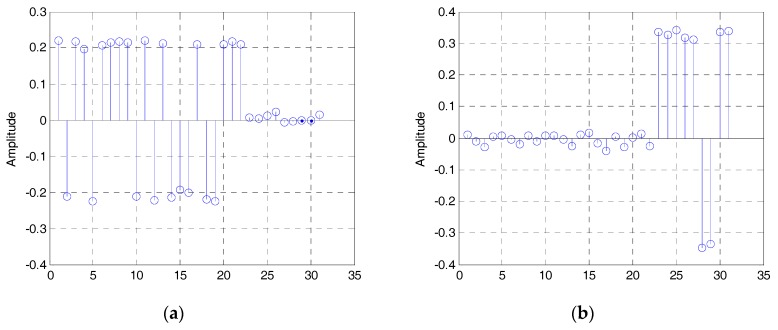
The estimated eigenvectors: (**a**) The eigenvector corresponding to the largest eigenvalue; (**b**) the eigenvector corresponding to the second largest eigenvalue.

The estimated eigenvalues of the autocorrelation matrix of the DSSS signal are shown in Figure 3, and the normalized eigenvectors $e_{1}$, $e_{2}$. corresponding to the largest and second largest eigenvalues are shown in Figure 4a,b. Both of them are estimated by the modified LEAP (α=0.9,γ=2) and the SNR is −5 dB.

**Figure 5:**
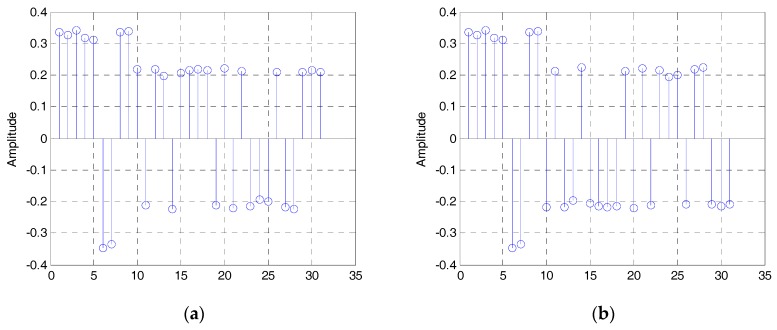
The estimated sequences: (**a**) $b_{1}$; (**b**) $b_{2}$.

**Figure 6:**
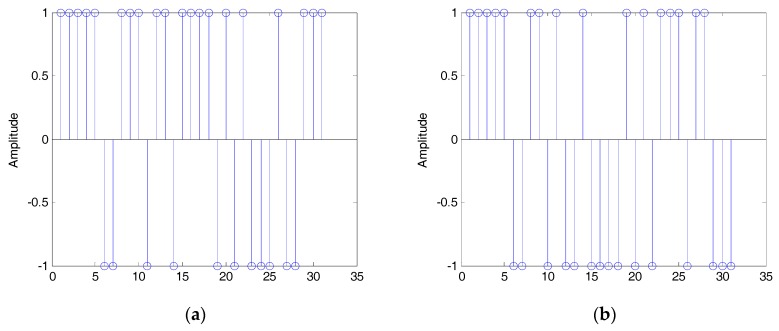
The estimated PN sequences: (**a**) ${\hat{PN}}_{1}$; (**b**) ${\hat{PN}}_{2}$.

Then, the two eigenvectors shown in Figure 4 are concatenated. Because the phase of eigenvectors can be reversed, we can get two different estimated sequences $b_{1}$ and $b_{2}\;$. shown in Figure 5 (here, we regard b and −b as the same). Then, according to Equation (27), the estimated PN sequences are:

(34) $${\overset{⌢}{PN}}_{i}=\mathrm{sgn}\left(b_{i}\right),i=1,2,$$

which are shown in Figure 6. On the basis of Equation (29), in this simulation, $\psi_{1}=102$, $\psi_{2}=94$. Since $\psi_{1}>\psi_{2}$, the true estimated PN sequence is ${\hat{PN}}_{2}$, which is the same as the true PN sequence of the DSSS signal shown in Figure 2. By now, the above simulation results show the validity of the proposed method, even with very low SNR.

**Figure 7:**
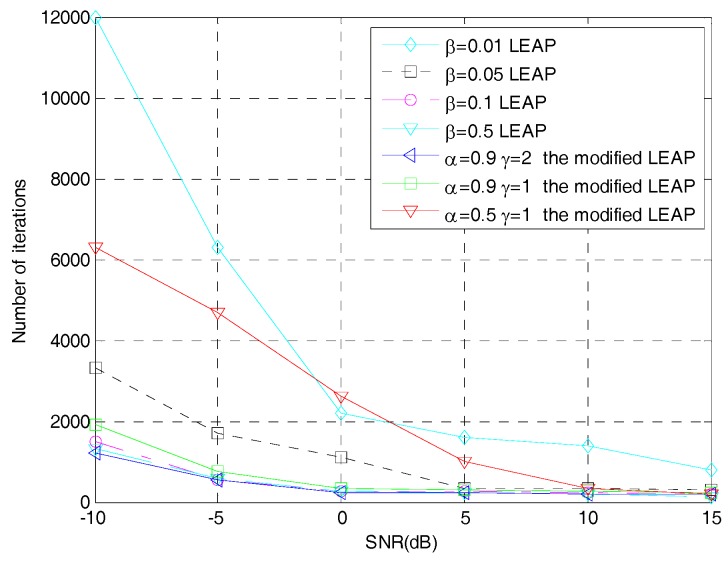
Number of iterations for network convergence of the original LEAP and the modified LEAP sunder different SNRs.

**Figure 8:**
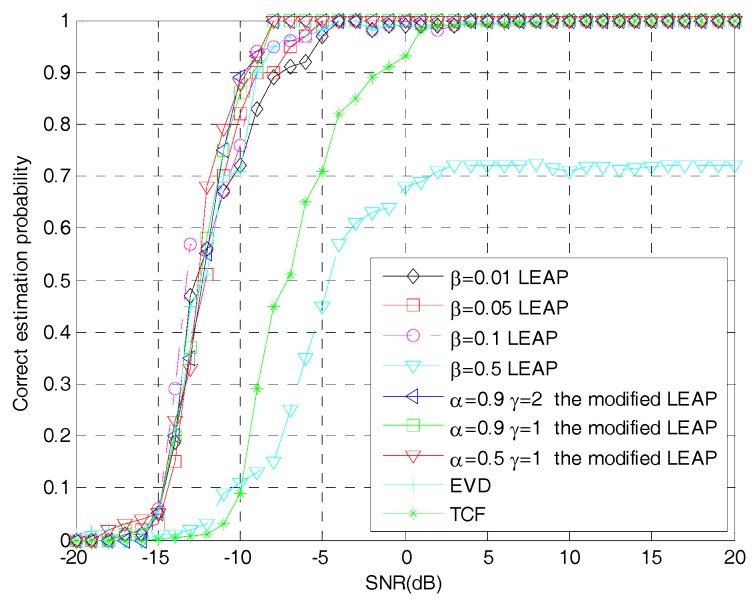
Correct PN sequence estimation probability of different methods under different SNRs.

Figure 7 shows the relationship between the number of iterative steps required by the modified LEAP and the original LEAP to make the network converge at different learning steps as the SNR changes. Figure 8 shows the relationship between the correct estimation probability of the PN sequence and SNRs, when using the modified LEAP and the original LEAP at different learning steps as well as the TCF- and EVD-based methods. Both tests use 1000 Monte Carlo simulations. 

It can be seen from Figure 7 and Figure 8 that when the learning step is set to β=0.01 and β=0.05 in the original LEAP, the network can stably converge to the optimum point due to the small step size, but the number of iterations required for network convergence is relatively large, which is similar to the modified LEAP when α=0.5,γ=1. When the learning step is set to β=0.5 in the original LEAP, although the large learning step increases the convergence speed of the network, the correct estimation probability of the PN sequence is seriously reduced, because the network cannot converge to the optimum point. When the learning step of the original LEAP is set to β=0.1, the original LEAP can not only converge rapidly, but also get good estimation performance, which is similar to the modified LEAP when α=0.9, γ=1 or α=0.9, γ=2. However, in practical applications, it is difficult to choose a suitable learning step when using the original LEAP, but in the modified LEAP, you just need to set the weight coefficients α and γ slightly larger, then the network will first approach the optimum point with a large step size and get to the optimum point with a small step size, which can not only make the network converge rapidly, but also obtain a high correct estimation probability of the PN sequence of the DSSS signal. Moreover, according to Figure 8, it is obvious that when the LEAP-based methods can converge correctly, their performance is comparable to that of the EVD-based method and is superior to that of the TCF-based method. The reason is twofold. First, since the LEAP neural network is actually a principal component analysis network, the principle of the LEAP-based methods and the EVD-based method is the same, which means that their performance is comparable. Second, when using the TCF-based method for PN sequence estimation, the peaks of the TCF need to be accurately searched [8], which is difficult to achieve in low SNR environment. Therefore, the TCF-based method has poor performance when the SNR is low.

## 6. Conclusions

A blind PN sequence estimation method of the DSSS signal using a modified LEAP is proposed in this paper. Compared to the original LEAP, the modified LEAP makes it easier to set the suitable learning step size to obtain good convergence performance. When the modified LEAP is converging, the PN sequence of the DSSS signal can be estimated by the connection weights of the network. These weights are the eigenvectors of the autocorrelation matrix of the DSSS signal. Because of the phase ambiguity of the eigenvectors, a novel approach which is based on the characteristics of the PN sequence of the DSSS signal is also proposed here to exclude the wrong estimated PN sequences. As shown in the simulations, the proposed methods mentioned above can quickly estimate the PN sequence of the DSSS signal in a low SNR environment.

## 7. Patents

F.S.; Y.W.; X.W.; C.Z. PN sequence estimation of DSSS Signals based on a variable step size online unsupervised learning machine. China Patent 201810861275.2, 1 August 2018.
